# Optimised electronic patient records to improve clinical monitoring of HIV-positive patients in rural South Africa (MONART trial): study protocol for a cluster-randomised trial

**DOI:** 10.1186/s12879-021-06952-5

**Published:** 2021-12-20

**Authors:** Collins Iwuji, Meg Osler, Lusanda Mazibuko, Natalia Hounsome, Nothando Ngwenya, Rujeko Samanthia Chimukuche, Thandeka Khoza, Dickman Gareta, Henry Sunpath, Andrew Boulle, Kobus Herbst

**Affiliations:** 1 Department of Global Health Infection, Brighton and Sussex Medical School, University of Sussex, Falmer, Brighton, BN1 9PX UK; 2 Africa Health Research Institute, Durban, KwaZulu-Natal South Africa; 3 Centre for Infectious Disease Epidemiology and Research, School of Public Health and Family Medicine, University of Cape Town, Cape Town, South Africa; 4 Nelson R Mandela School of Medicine, University of KwaZulu-Natal, Durban, South Africa; 5 Department of Health, Provincial Government of the Western Cape, Cape Town, South Africa; 6 DSI-MRC South African Population Research Infrastructure Network, Durban, South Africa

**Keywords:** HIV, Viral load monitoring, Virological failure, Drug resistance, Viral load champion

## Abstract

**Background:**

There is poor viral load monitoring (VLM) and inadequate management of virological failure in HIV-positive individuals on antiretroviral therapy in rural KwaZulu-Natal, South Africa. This could be contributing to increasing HIV drug resistance in the setting. This study aims to investigate the clinical and process impediments in VLM within the health system and to evaluate a quality improvement package (QIP) to address the identified gaps. The QIP comprises (i) a designated viral load champion responsible for administrative management and triaging of viral load results (ii) technological enhancement of the routine clinic-based Three Interlinked Electronic Register (TIER.Net) to facilitate daily automatic import of viral load results from the National Health Service Laboratory to TIER.Net (iii) development of a dashboard system to support VLM.

**Methods/design:**

The study will evaluate the effectiveness of the QIP compared to current care for improving VLM and virological suppression using an effectiveness implementation hybrid type 3 design. This will use a cluster-randomised design with the primary healthcare clinics as the unit of randomisation with ten clinics randomised in a 1:1 ratio to either the intervention or control arm. We will enrol 150 HIV-positive individuals who had been on ART for ≥ 12 months from each of the ten clinics (750 in 5 intervention clinics vs. 750 in 5 control clinics) and follow them up for a period of 12 months. The primary outcome is the proportion of all patients who have a viral load (VL) measurement and are virally suppressed (composite outcome) after 12 months of follow up. Secondary outcomes during follow up include proportion of all patients with at least one documented VL in TIER.Net, proportion with VL ≥ 50 copies/mL, proportion with VL ≥ 1000 copies/mL (virological failure) and subsequent switch to second-line ART.

**Discussion:**

We aim to provide evidence that a staff-centred quality improvement package, designated viral load monitoring champion, and augmentation of TIER.Net with a dashboard system will improve viral load monitoring and lead to improved virological suppression.

*Trial registration:* This trial is registered on ClinicalTrials.gov on 8 Oct 2021. Identifier: NCT05071573; https://clinicaltrials.gov/ct2/show/NCT05071573?term=NCT05071573&draw=2&rank=1

## Background

Antiretroviral therapy (ART) scale-up in sub-Saharan Africa (SSA) has been rapid with an estimated 16.4 million individuals on ART by the end of 2018 [1]. The increased expansion in the indication for ART use would result in earlier initiation of treatment and if adherence is suboptimal, could lead to virological failure and the likely development of drug resistance. The likelihood of transmission of resistant virus will depend on ART coverage, duration of ART roll-out and the proportion and absolute numbers of individuals with virological failure [2–4]. Conditions that would influence transmission of resistant virus include proportion of failures with resistant virus, time spent on failing regimen, viral load (VL) of patients with resistant virus, fitness of the resistant virus and the transmission probability compared with ART-naïve individuals [2]. The availability of viral load monitoring (VLM) in resource-rich countries, means individual failing treatment are identified early and switched to alternative suppressive ART. In contrast, many sub-Saharan African countries rely on World Health Organisation (WHO) immunological and clinical criteria for identifying treatment failure which has poor sensitivity and specificity [5]. In settings where there is availability of VLM, their effectiveness has been hampered by poor adherence to monitoring guidelines by healthcare providers [6].

South Africa has the biggest HIV treatment programme in the world with 7.7 million individuals living with HIV and 62% currently receiving ART within a stretched health system [1]. VLM has been part of the public ART programme since roll-out in 2004 and requires people living with HIV (PLHIV) initiating ART to have a VL measured at 6 months and 12 months after ART initiation and 12-monthly thereafter if virologically suppressed [7, 8]. Those with a VL ≥ 1000 HIV-1 RNA copies/mL should be retested after 3 months of adherence counselling support, and then either retained on first-line therapy if re-suppressed or switched to second-line therapy if VL ≥ 1000 copies/mL [7].

However, little is known about how these VL guidelines are being used in clinical decision-making in public ART programmes in SSA. In formative research utilising an electronic database, the Three Interlinked Electronic Register (TIER.Net) [9], of a programmatic ART cohort in rural KwaZulu-Natal, we observed infrequent VLM and sub-optimal management of virological failure. The study showed that only 34% of patients had a viral load documented after 12 months on ART. Only 20% of individuals in the cohort were confirmed to have virologic re-suppression or change to second line therapy after virologic failure, and those who changed therapy did so a median of one year after virologic failure [10]. With the expansion in the indications for ART use [11], such delays are likely to have significant deleterious individual and public health impacts through effects on patient morbidity, accumulation of drug resistance [12–14], and persistent risk of HIV transmission in the setting [15].

Findings from this formative research was presented in a series of workshops to healthcare providers, policy makers, researchers and community representatives using good participatory approaches with a view to co-produce health system interventions to address poor VLM. This protocol describes the proposed interventions and their evaluation. We hypothesise that a staff-centred quality improvement package (QIP) and technological augmentation of an existing electronic ART database (TIER.Net) would result in optimal VLM of patients on ART, prompt clinical management of virological failure and an overall improvement in virological suppression.

## Objectives

### Main trial objective

The main objective is to evaluate the impact of a combination of interventions that includes a staff-centred quality improvement package, designated viral load monitoring champion, and augmentation of TIER.Net with a dashboard system will improve viral load monitoring and lead to better virological suppression over a period of 12 months in comparison to the current standard of care.

### Secondary objectives

- To identify health system specific gaps in VLM.
- To evaluate the cost and cost effectiveness of the intervention compared to standard care
- To undertake a process evaluation assessing acceptability, fidelity, adaptation and contexts in the implementation of the intervention.

## Methods

### Trial setting

The trial will be hosted by the Africa Health Research Institute (AHRI), an independent scientific research institute and a Wellcome Trust Africa and Asia Programme. AHRI has two campuses 250 km apart; the Population Intervention Programme (PIP) study area in uMkhanyakude district in northern KZN with a population of 625,846, and a state-of-the-art laboratory infrastructure in the city of Durban.

The PIP is an extensive social, demographic, HIV and clinical research programme of an under-resourced rural population of 140,000 in an area of about 845 km^2^. Hlabisa is at the epicentre of the HIV epidemic in South Africa, where HIV prevalence among men and women aged 15–54 years in 2018 was 19% and 40% respectively [16]. It is situated in the most economically deprived district nationally; about 5% of adults have completed a higher education, 4% are covered by a medical aid scheme and unemployment rate is 62% [16]. AHRI has a memorandum of understanding with the DoH to support HIV care in the Hlabisa sub-district with ~ 30,000 PLHIV currently on ART (still in active care) in the 17 primary healthcare clinics (PHC) in the catchment area. This trial will be implemented in 10 of the 11 primary health care clinics that are located within the catchment area of the PIP.

The study involves collaboration of investigators from AHRI, the University of Sussex (UoS) and the University of Cape Town (UCT).

### Trial design

#### Gap analysis

We will use a retrospective chart review of HIV positive patients in care at the 10 trial clinics to identify the health systems gaps in VLM. We will create sampling frame of individuals who are in care and started ART 15–18 months previously and select a random sample of 800 individuals from this frame. Clinical notes of these individuals will be reviewed to identify any gaps in the VLM process, and to identify predictors of missing viral loads. This information will be used to develop a QIP that includes training of staff on the current South African ART guidelines, the importance of VLM and both health system and individual risk factors for suboptimal VLM.

#### Evaluation of the interventions

**Fig. 1:**
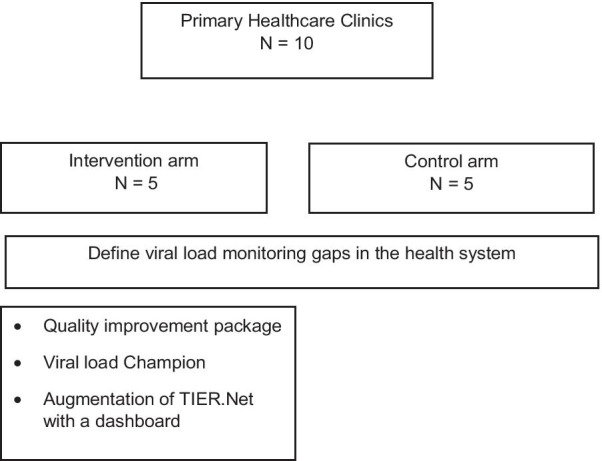
Trial design

We will use an innovative effectiveness-implementation hybrid cluster-randomised trial in the 10 clinics to evaluate the effectiveness of the interventions for improving VLM and virological suppression compared with current care (Fig. 1). Clinics will be randomly allocated to either the intervention (QIP, viral load champion, and augmentation of TIER.Net) or current care.

### Participants

Gaps in VLM will be identified in HIV-positive patients aged ≥ 16 years who started ART in the previous 15–18 months in any of the 10 trial clinics through a retrospective review of their clinical charts.

The intervention will be evaluated in all patients aged ≥ 16 years who have been on ART for ≥ 12 months during the study’s baseline time period and attend any of the 10 trial clinics during a three-month recruitment period. No exclusion criteria will apply as there will be no individual enrolment to the trial, rather routine clinical data will be used for the evaluation of the outcomes.

### Description of the intervention

#### Quality improvement package

We will adapt the VL champion model described by Sunpath et al. [17]. This model identifies a VL champion in each clinic who dedicates 10 h per week to the role. The VL champion ensures those with high VL are appropriately managed with repeat VL tests including referral to a doctor for second line ART switch if required.

The prototype QIP will be adapted based on the identified clinical and process impediments in VLM from the gap analysis. All nursing/assistant staff in the intervention clinics will be trained on the QIP using the model by Sunpath et al. as described above [17]. Each intervention clinic will identify two key nurses who would act as VL champions. Their role will include tracing and recalling patients who need further assessments of their VL or switch to second-line regimen.

Training will be provided in the use of enhanced TIER.Net technology that will be developed as part of the trial and how to access reports on the dashboard system.

#### Augmentation of TIER.Net with a dashboard system

TIER.Net is an electronic database with varying levels of sophistication in both functionality and reports. It ranges from an off-line version of the electronic register to hybrid that is predominantly offline, with synchronisation centrally when bandwidth allows to a networked electronic version of the paper register [9]. The offline version of TIER.Net is in operation in the public ART programmes in which this study will be embedded.

As part of the proposed intervention, VL results will be imported into TIER.Net daily from the National Health Laboratory Service (NHLS) which will be linked to patients in TIER.Net based on multiple exclusive and linked deterministic rules using a combination of variables such as name, surname, sex, date of birth, date of visit, NHLS lab number, facility and folder number. The information contained in TIER.Net will be used to develop a dashboard which will display the following information:

- The proportion due VL tests as per South African ART guidelines.
- The proportion with high VLs (VL ≥ 50 copies/mL).
- The proportion lost to follow-up.
- Those with confirmed virological failure (repeat VL ≥ 1000 copies/mL after a first VL 1000 copies/mL measured ≥ 3 months apart).

A dashboard has the advantage of displaying a quick visual state of affairs and how this evolves with time. This will incorporate a text messaging functionality that can be used by healthcare workers/viral load champion to communicate with patients.

It is envisaged that these interventions will result in prompt identification of patients who require VLM, and improve clinical management of virological failure thereby improving the quality and efficiency of service delivery.

### Standard of care

Viral load results are manually captured on to the TIER.Net system with filing of the paper results in patients’ clinical notes for nurses’ review during routine appointments. This is used to produce a monthly enrolment and quarterly cohort reports for the central monitoring of the ART programme.

### Outcome measures

#### Health system gaps

- Proportion of patients in survey sample with VL documented in clinical charts but not in TIER.Net
- Proportion of patients with VL documented in TIER.Net but no results in clinical charts
- Proportion of patients with no VL in chart or TIER.Net (true missing VL = no assessment of VL undertaken)
- Predictors of true missing viral load (no record in either TIER.Net or clinical charts)

#### Evaluation of intervention

##### Primary outcomes

Proportion of all patients who have a VL measurement and are virally suppressed (composite outcome) after 12 months of follow up.

##### Secondary outcomes

- Proportion of all patients with at least one documented VL in TIER.Net during the trial follow up.
- Proportion with VL ≥ 50 copies/mL during follow up
- Proportion with VL ≥ 1000 copies/mL during follow up (virological failure)
- Among patients with VL ≥ 1000:
  - Proportion with a repeat test within 3 months
  - Time from first VL ≥ 1000 copies/mL to repeat VL.
  - Proportion switching to second-line ART after two consecutive VL ≥ 1000 copies/mL measured ≤ 3 months apart
  - Proportion completing the treatment failure cascade (VL < 50 copies/mL on repeat testing or switch to second line ART)
- Cost-effectiveness of the intervention compared to current care with respect to the primary outcome (viral suppression at 12 month follow-up) and two secondary outcomes (proportions of patients with VL ≥ 50 copies/mL and VL ≥ 1000 copies/mL)
- Acceptability, fidelity, adaptation and contexts in the implementation of the interventions

### Sample size justification

#### Health system gaps

The precision with which the prevalence of each outcome can be estimated will depends on the prevalence itself and the amount of correlation within clinics (the ‘design effect’). For a prevalence of 30%, a sample size of 800 patients will allow us to estimate it with a precision of ± 4.5 or ± 5.5% with 95% confidence if the design effect is 2 or 3, respectively. For a prevalence of 60%, the corresponding figures are ± 4.8 and ± 5.9%. This sample size will also allow us to determine the association of factors with a missing VL measurement. For example, if the design effect is 2, assuming the prevalence of missing VL is between 30 and 60%, we would have > 80% power to detect an OR of 2.25 or more for factors associated with missing VL, if the prevalence of the risk factor among those with non-missing VL is between 15 and 70%. This would allow us to identify any strong predictors of missing VL.

#### Evaluation of the intervention

We estimate that during the 3 month recruitment phase, an average of 150 patients who have been on ART for ≥ 12 months will attend each clinic. Based on our previous work, we expect that around 50% of patients in the control arm will have a VL measurement and be virally suppressed at the end of follow-up. With 5 clinics per arm, assuming a coefficient of variation, k, of 0.20, and a sample size of 150 patients per clinic, we would have 80% power to detect an increase in the proportion of patients who have a VL measurement and are virally suppressed from 50% in the control arm to 77% in the intervention arm (Table 1).

**Table 1:** Numbers needed to demonstrate an improvement in viral load monitoring, for different assumptions regarding clinic size and effect sizes

Number of clinics per arm	Number of patients per clinic^1^	Coefficient of variation (k)	% with VL suppressed in control	% with VL suppressed in intervention	Power (%)
5	100	0.20	45	67	71
5	100	0.20	45	70	80
5	100	0.20	50	74	71
5	100	0.20	50	77	80
5	100	0.20	55	80	70
5	100	0.20	55	84	80
5	150	0.20	45	66	70
5	150	0.20	45	69	80
5	150	0.20	50	73	70
5	150	0.20	50	77	80
5	150	0.20	55	80	70
5	150	0.20	55	84	80

^1^Harmonic mean to account for varying facility size

### Recruitment

#### Health system gaps

We will create sampling frame of all HIV-positive patients who are in care at the ten trial clinics and who started ART in the previous 15–18 months. We will select a random sample of 800 individuals above the age of 16 years from this list. The clinical notes of these individuals will be reviewed to identify any gaps in the viral load monitoring process.

#### Evaluation of the intervention

The effectiveness of the intervention will be evaluated in HIV-positive individuals aged ≥ 16 years attending the trial clinics who have been on ART for at least 12 months at baseline (the evaluation cohort).

AHRI has a clinical research assistant stationed in each of the ten clinics who registers every patient attending the clinic, irrespective of their reason for attendance or HIV status. A database, known as ClinicLink, was developed in-house at AHRI for the registration of clinic visits and reasons for attendance. ClinicLink will be used to identify patients attending the clinic who meet the criteria for inclusion in the evaluation cohort. Based on current clinic attendance, we will need about three months to meet the target sample size of an average of 150 patients per clinic. These individuals will form the basis for the main analyses. In secondary analyses, all patients meeting these criteria who attend one of the trial clinics during the three-month recruitment phase will be included in the evaluation cohort, even if the sample size is exceeded since the target sample size is considered a minimum. If the target sample size is not met within 3 months, we will extend the recruitment phase for another month as necessary.

### Allocation and blinding

The 10 clinics will be randomly allocated to the intervention (5 clinics) and control (5 clinics) arms. Randomisation will be stratified on clinic size, defined by the number of HIV-related visits per month (2 strata). Restricted randomisation will be used to ensure balance between study arms on the following important covariates: proportion of HIV patients who are male; proportion of HIV patients who are aged < 25 years. A computer programme will be used to prepare a list of all permissible randomised combinations; community leaders will be invited to make a random selection from the list at a public ceremony. The randomisation list will be prepared by an independent AHRI statistician and will be concealed until after randomisation. It will not be possible to blind the research staff and nurses to the intervention.

### Data collection

A secure Research Electronic Data Capture (REDCap) [18] project will be developed that will enable consistent data entry online using mobile connectivity. Standardised questionnaires will be used to collect data electronically in a robust fashion enabling patients to be identified through unique identifier or combination of demographic information such as names, surnames, date of birth, sex and other personal identifiers (e.g. South African identity number) already captured in TIER.Net database.

Before data collection begins, the REDCap will be thoroughly tested and subjected to a range of checks culminating in the entry of data for a group of ‘dummy’ patients, and this will be followed by extraction of dummy dataset by a research data manager and derivation of key study outcome measures by the study statistician to ensure data fields and response coding are clear. After all checks, the database is ‘signed off’ by the research data manager and statistician.

Once data entry has begun periodic validation and management checks will be performed using Pentaho Data Integration to assess data completeness, rate of recruitment and data consistency. Exception reports will be created, and issues raised will be queried with the study coordinator.

#### Health system gap

The following data will be extracted from chart review into REDCap to document if (i) viral load was actually not measured as evidenced by a lack of documentation of this in the clinical charts and in TIER.Net (ii) there was documentation of a blood draw for VL and the date of the test (iii) results were filed and entered into the patient charts although not captured in TIER.Net (iv) there was evidence in patient chart that test was done but no results filed (v) VL results and date present in TIER.Net but not filed in patient charts.

All data collected from clinical chart reviews will be centrally stored in a master MONART database located at Africa Health Research Institute. This database will integrate data collected from clinical charts reviews, hospital information management system (HIS), ClinicLink and TIER.net. Database rules and constraints will be added and enforced on the master database to ensure that only valid data is stored in the database.

A study dashboard will be developed to assist the operational teams monitor the progress of the study.

#### Evaluation of the intervention impact

TIER.Net has information on HIV-positive individuals on ART including unique South African Identification number, age, sex, date of ART initiation, viral load results and dates, CD4 count results and dates, type of ART regimen, status of the individual (whether still in care, lost to follow up or dead), and dates of clinic appointments. The clinical charts of the 150 participants per clinic who are in the evaluation cohort will be reviewed to extract these baseline data for entry into REDCap. These will be repeated after each participant has completed 12 months of follow up in their clinic/cluster to capture documentation of VL being measured, test data and the results of the VL. These data will be used to evaluate the impact of the intervention on VLM and virological suppression, and on the treatment failure cascade. Data on all clinic visits irrespective of reason for the visit will be extracted from ClinicLink. These will be used to describe patterns of health care use among the trial population. HIV-positive individuals receiving care in the clinics are not the recipients of the quality improvement interventions, which will be focussed on the nurses and systems for delivering care. Hence, interim visits for any intercurrent illness by patients will be managed as per standard clinical care and will not be routinely reported as adverse events.

### Trial procedures and timelines

**Fig. 2:**
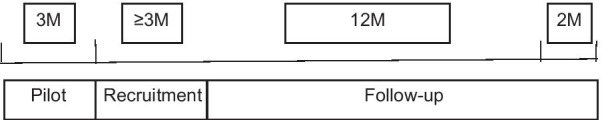
Study timelines

We will pilot the interventions over 3 months to address any issues raised and deliver further training to the nurses or adaptation of the technology if required (Fig. 2). Patients attending for their routine clinic visits over a three month period, which we estimate is the time required for at least 150 participants to pass through each clinic, will be included in the evaluation cohort and followed up for a period of 14 months to allow a 2 month window for appointment reschedules etc.

### Data management

Due to data protection regulation, AHRI will act as data controller for the project. UCT and UoS will be data processors but will only process anonymised data for the purpose of analysis. It will be the responsibility of the data custodian based at AHRI to ensure that data access is consistent with terms of the AHRI Data Access Policy and ethical approval obtained for this protocol.

Data will be stored on industry-standard relational databases with data integrity and user authentication for access control. Data will be replicated on at least a daily basis to the Durban site of the Institution to provide secure offsite storage of data. Transactional logs will be backed up every 30 min to enable recovery of data in the event of equipment failure.

All users of the MONART electronic data collection system (REDCap) and database will be authenticated through individual passwords with minimum complexity and regular change rules (passwords must be at least eight characters, with a mix of small and capital letters, at least one numeric or non-alphabetic digit and changed at least every 45 days). The Institution use industry standard malware and intrusion detection with regular penetration tests by a reputable external security audit company.

Both at the Institute and for the clinic-based data collection, a client–server architecture will be implemented where data is not stored on laptops or local workstation, but only on a central server with restricted physical access. Specifically, at the clinics the local server will be enclosed in a tamper-proof enclosure kept under lock and key.

Once data have been stored in the MONART databases, they will become the responsibility of the head of research data management. Research data management will regularly assess data quality and implement corrective measures to ensure data quality. At the end of the study, anonymised analytical datasets will be created, documented and archived on the AHRI data repository. A digital object identifier (DOI) will be created and added on the dataset documentation for reference purpose. Research data management will administer a data repository that will contain all datasets required for analysis for the trial. They will ensure that the repository has access control measures that enforce the data access criteria determined by the study data steering committee. Access to the datasets will be granted to those who meet minimum requirements set out by the study data steering committee.

All correspondence relating to this project will be kept in appropriate file folders. Any confidential patient or site information will be kept in secure, limited access locations. Records of participants, other source documents, pertaining to the study will be kept on file for a period of 10 years per South African Good Clinical Practice guidelines (SA GCP).

### Statistical analysis

#### Analysis of health system gaps

Analysis of data from the retrospective chart review of patients will quantify clinical and process impediments in VL testing system; results will inform the VL testing pathway, a QIP and required augmentation of TIER.Net. Proportions of patients with outcomes of interest will be tabulated; 95% confidence intervals will be calculated using clustered standard errors to take correlation within clinic into account. Factors associated with true missing viral load (not documented in TIER.Net or present in the patient chart) will be analysed using logistic regression with robust standard errors.

#### Evaluation of the trial intervention

The effect of the intervention on the primary outcome (proportion of patients with VL suppression) will be estimated using a two-stage approach based on cluster (i.e. clinic)-level summaries [19]. The cluster-level approach, although less statistically efficient than methods based on individual level regression, is more robust when there are a relatively small number of clusters. Briefly, in the first stage, the proportion with VL suppression in each clinic will be calculated, and logarithmically transformed. In the second stage, the cluster-level summaries will be compared using a stratified t-test to test the null hypothesis of no intervention effect. Since equal numbers of clinics will be allocated to the 2 arms within each stratum, the unadjusted risk ratio (RR) for the intervention effect will be calculated as the ratio of geometric mean proportion with VL suppression for the 5 clusters in each arm. The 95% confidence interval (CI) will be calculated with the variance estimated from the residual mean square in a two-way analysis of variance of the log proportions on stratum and trial arm, incorporating a stratum by trial arm interaction. An adjusted analysis based on cluster-level summaries may also be done to account for covariate imbalance at baseline, using a similar two-stage process. All covariates will be pre-specified in a detailed analysis plan. In the first stage, an individual-level logistic regression will be done, including terms for stratum and all covariates except the intervention effect, and ignoring clustering. After fitting the regression model, a residual for each cluster will be calculated as the ratio of the observed to the predicted number of events. If the intervention has no effect, the residuals of the two arms should be similar on average. In contrast, if the intervention has an effect, we expect the residuals to differ systematically. Therefore, in the second stage, we will compare the residuals between the trial arms using the methods described above; residuals will be log transformed for the analysis. Analysis of the intervention effect on the secondary outcomes will be done using the same approach. A detailed statistical analysis plan will be prepared before to data analysis.

### Trial oversight

It was not relevant to set up a data monitoring and safety board as routine health data will be assessed for study outcomes following the quality improvement intervention delivered to research nurses and augmentation of TIER.Net. Hence, we decided to set up a trial steering committee in addition to the trial management group.

#### Trial management group (TMG)

The trial management group will include the principal investigator and all the co-investigators including study statistician, research data manager, study coordinator and the operational managers of the five intervention clinics. The TMG will be chaired by the principal investigator and will have day to day oversight responsibility for the conduct of the trial.

They will meet once a month either in person or virtually to discuss the progress of the trial. They will also review all data from the trial and will be the main decision making body of the trial. They will be advised by an independent trial steering committee.

#### Trial steering committee (TSC)

This will consist of individuals who are independent from the study team. They will be joined by members of the TMG including the principal investigator, an investigator nominated by each collaborating institution, study statistician, study coordinator and research data manager. The composition will be specified in the TSC charter. The TSC will have operational responsibility for the overall conduct and management of the trial. The group’s terms of reference, roles and responsibilities will be defined in a standard operating procedure. One of the independent members of the TSC will act as the chair.

### Research ethics and consent

This study is registered with Clinicaltrials.gov (NCT05071573). The main study findings will be reported in accordance the Consolidated Standards of Reporting Trials (CONSORT) statement [20]. The study protocol v1.1, 19 Aug 2020 received ethical approval from the Biomedical Research Ethics Committee of the University of KwaZulu-Natal (BREC/1531/2020), the Research Governance and Ethics Committee of the Brighton and Sussex Medical School (ER/BSMS9B5G/4) and the KwaZulu-Natal Health Research Committee (KZ_202008_092). Any protocol amendments will need to be approved by all regulatory authorities mentioned above prior to implementation. Research staff will be trained in the amended protocol and any updated study materials and consent forms will be administered to the relevant participants as necessary. The University of Sussex is the sponsor of the study.

The operational managers of the 10 trial clinics will be given clear explanations about the trial aims, and the nature, scope, and possible consequences of their facilities and staff participating in the research by the study coordinator. Managers will be asked to provide written informed consent for their facilities to participate in the trial and be randomised to one of the two trial arms, and for review of clinical records of patients.

The principal investigator and study coordinator will schedule meetings with nursing staff from the 10 clinics, to explain the intervention and the trial aims. Nurses will be asked to consent to participation in the trial, and to receive further training as viral load champions if selected. They will be offered consent for participation in the process evaluation (not described in this protocol).

The informed consent process will be administered by a research assistant through REDCap with electronic signature obtained from nurses participating in the QIP. The nurses will be given paper copies of the participant information leaflets and consent forms.

A waiver of individual written consent from patients for a review of their clinical records is approved by all the regulatory authorities mentioned above. We requested this waiver for the following reasons: (1) the research involves minimal risks to the patients, (2) the extracted data will be anonymised hence there is no risk of the confidentiality of patients being compromised, and (3) analytical results will be reported at population or group level and not at the level of the individual patient.

We have a memorandum of agreement with the Department of Health to provide access to records in TIER.Net. Extraction of data from TIER.Net and ClinicLink, and linkage to other AHRI databases, has been previously approved by the University of KwaZulu-Natal Biomedical Research Ethics Committee (Ref: BE290/16).

### Data protection and patient confidentiality

All investigators and trial site staff will comply with the requirements of the Protection of Personal Information Act (or POPI Act) [21] which is the South African equivalent of the General Data Protection Regulation 2018 [22] with regards to the collection, storage, processing and disclosure of personal information and will uphold the Act’s core principles. Personal information will be collected, kept secure, and maintained. This will involve the creation of coded, depersonalised data where the participant’s identifying information is replaced by a unique identifier and the linking code is kept in separate locations using encrypted digital files within password protected folders and storage media.

### Dissemination

Research findings will be fed directly back to the community advisory board and the district and provincial departments of health prior to sharing this with the wider scientific community. In addition, under the existing memorandum of agreement between AHRI and the KwaZulu-Natal Department of Health, all scientific work (manuscript, conference abstracts) will be submitted for review by the department of health through the Head of the Health Research Committee. Study staff will feed results back to sites involved in collaboration with the department of health as they emerge and discuss areas that require strengthening. Investigators will publish findings in peer-reviewed journals and present at relevant conferences and meetings. Access to the final dataset will be according to AHRI data sharing policy available at https://data.ahri.org/index.php/home. Authorship of research manuscripts will follow the International Committee of Medical Journal Editors guidelines [23].

## Discussion

In the formative research leading up to the interventions proposed for evaluation in this protocol, we showed poor viral load monitoring and inadequate management of virological failure in HIV-positive patients on ART in rural KwaZulu-Natal, South Africa. Non-Nucleoside reverse transcriptase pre-treatment drug resistance exceeds 10% in the setting [24]; the threshold required to trigger a change in first-line ART using the public health approach [25]. In response to this, the South African ART guidelines now recommends dolutegravir, an integrase inhibitor in place of an efavirenz-based first-line ART [26]. Poor VLM may shorten the therapeutic lifespan of dolutegravir-based first-line ART due to development of resistance and predispose to poor clinical outcomes in HIV-positive individuals in public ART programmes in sub-Saharan Africa ([27]. Drug resistance is associated with increased morbidity and mortality with the risk of transmitting drug-resistant HIV to sexual partners.

The observed poor VLM in KwaZulu-Natal could be due to a number of reasons; patients may not have been aware of or empowered to request recommended blood tests, facility staff may be inadequately trained, or have insufficient time or resources to conduct blood tests when due, failure of results to reach clinics from the laboratory or failure to capture results on TIER.Net as these are manually entered [10]. The current study proposes to investigate the clinical and process impediments in VLM, including a review of patients’ clinical charts and comparing viral load in the charts with viral load data manually captured on TIER.Net in order to clarify whether the reported poor VLM was due to poor data entry in TIER.Net or actual failure to monitor viral loads. The proposed interventions will address all the steps potentially implicated in the poor management of VLM. This will involve training of staff on the current South African ART guidelines [26], designating a viral load champion who has administrative responsibility for identifying individuals due viral load tests or those with confirmed virological failure who need to be switched to second-line ART [17]. The automatic import of viral load results from the NHLS laboratories, also eliminates staffing challenges which contributes to backlogs in data capture and data entry errors. We also observed that amongst those with VLM who are experiencing virological failure, there is poor health system response to the clinical management of this situation. Only a minority of those with elevated viral load had a repeat viral load to confirm virological failure on ART, with only a small proportion of those with confirmed virological failure switching to second-line ART [10].

Hence there is urgent need to evaluate interventions that will improve VLM and the management of virological failure in ART programmes in sub-Saharan Africa. These interventions will have to be cost-effective, acceptable by patients and feasible to adopt in the health system. These additional important objectives will be addressed in this research but are not covered in this protocol. If the combination of interventions proposed for evaluation in this trial demonstrates effectiveness, the resulting early detection and prompt management of virological failure will be critical towards achieving UNAIDS 95-95-95 (95% of people living with HIV aware of their diagnosis, 95% of those diagnosed on ART and 95% of those on ART being virally suppressed) targets by 2030 [28].

## Data Availability

Not applicable.

## Abbreviations

AHRI: Africa Health Research Institute
ART: Antiretroviral therapy
CI: Confidence interval
DOI: Digital Object Identifier
PIP: Population Intervention Programme
OR: Odds ratio
POPI: Protection of Personal Information
RR: Risk ratio
REDCap: Research Electronic Data Capture
TIER.Net: Three Interlinked Electronic Register
TSC: Trial steering committee
TMG: Trial management group
UCT: University of Cape Town
UoS: University of Sussex
QIP: Quality improvement package
UNAIDS: The Joint United Nations Programme on HIV/AIDS
VL: Viral load
VLM: Viral load monitoring
WHO: World Health Organisation
SSA: Sub-Saharan Africa
